# The UK National Homicide Therapeutic Service: A Retrospective Naturalistic Study Among 929 Bereaved Individuals

**DOI:** 10.3389/fpsyt.2020.00878

**Published:** 2020-08-28

**Authors:** Suzan Soydas, Geert E. Smid, Barbara Goodfellow, Rachel Wilson, Paul A. Boelen

**Affiliations:** ^1^Department of Clinical Psychology, Utrecht University, Utrecht, Netherlands; ^2^ARQ Centrum’45, ARQ National Psychotrauma Centre, Diemen, Netherlands; ^3^University of Humanistic Studies, Utrecht, Netherlands; ^4^ASSIST Trauma Care, Rugby, United Kingdom

**Keywords:** homicide, bereavement, loss, grief, posttraumatic stress disorder, bereavement interventions, psychological interventions

## Abstract

Homicidal bereavement puts survivors at risk of developing a broad range of lasting and severe mental health problems. Previous research has often relied on relatively small and homogenous samples. Still, little is known about what factors influence the expression of symptoms following homicidal bereavement. Preventive and curative treatments often do not consider the complex coherence between the emotional, judicial, financial, and societal challenges that likely arise following a homicide. Despite the severity of its consequences on mental health, no gold standard for the preventative and curative treatment of mental health issues in homicide survivors exists. We aimed to introduce a time-limited, traumatic grief-focused outreaching model of care designed specifically for homicide survivors, and to examine its potential effectiveness. Furthermore, we aimed to investigate what factors influence the severity of mental health problems and response to treatment. In the current study, self-reported data on five different outcome measures, namely, symptoms of posttraumatic stress, prolonged grief, depression, anxiety, and functional impairment were available from 929 homicidally bereaved treatment receiving adults. We used Latent Growth Modeling to analyze our repeated measures data and to classify individuals into distinct groups based on individual response patterns. Results showed that the current model of care is likely to be effective in reducing mental health complaints following homicidal bereavement. Having a history of mental illness, being younger of age and female, and having lost either a child or spouse consistently predicted greater symptom severity and functional impairment at baseline. For change in symptom severity and functional impairment during treatment, having a history of mental illness was the only consistent predictor across all outcomes. This study was limited by its reliance on self-reported data and cross-sectional design without a control group. Future prospective, longitudinal research across different cultures is needed in order to replicate the current findings and enhance generalizability. That notwithstanding, findings provide a first step toward evaluating a novel service-delivery approach for homicide survivors and provide further insight in the development of mental health complaints following bereavement by homicide.

## Introduction

In response to the death of a loved one, many people experience acute emotional distress and symptoms of mental health disorders, which generally decrease over time. However, for a significant minority, these symptoms may persist and develop into psychopathology, such as posttraumatic stress disorder (PTSD), major depressive disorder (MDD), anxiety disorders and prolonged grief disorder (PGD) [e.g., (61, 62)]. In recent literature on pathological grief reactions, PGD (1) is also referred to as persistent complex bereavement disorder (PCBD), a condition identified for further study in the DSM-5 [American Psychiatric Association (APA) (2)]. Because PGD is formally included in the ICD-11 [World Health Organization (WHO) (4)] and is related to PCBD (3, 4), in the current study the term PGD will be used.

Violent circumstances of the death constitute an important risk factor for developing psychopathology after loss (5). A well-known yet understudied example of violent death is homicide, which is defined by the WHO (4) as a fatal injury inflicted by another person with the intention to injure or kill. The term “homicide” covers the offences of murder and manslaughter (including corporate manslaughter) and infanticide (6). Every homicide is estimated to affect the lives of approximately 7 to 10 close relatives (7, 8), as well as those of numerous friends, neighbors, and co-workers. Those affected are often referred to as “homicide survivors” (9). Homicide survivors are typically underrepresented in media coverage, research reports, and government funds, that tend to focus on perpetrators (7, 10).

Research on the mental health of homicide survivors is relatively scarce, often involving homogenous and small samples (11–13). Studies suggest that homicide survivors are at elevated risk for developing PGD and at nearly doubled risk for developing PTSD, compared to other victims of violence and compared to individuals who were bereaved due to other causes (5, 14). Additionally, homicide survivors are prone to developing depression and anxiety symptoms, as well as feelings of guilt and responsibility. A study of over 400 families of homicide survivors showed that all participants reported that their mental health was somehow affected, with 25% of the survivors no longer working, 20% struggling with alcohol addiction, and 44% experiencing marital problems resulting in divorce or separation (10). Other studies reported problems such as eating disorders, insomnia (14, 15), sleep disorders, and suicide ideation (16, 17).

The high prevalence of mental health complaints in homicide survivors may be due to a number of complexities that are specific for bereavement following a homicide. Firstly, the shocking circumstances of the loss may induce intrusive images (18). Secondly, a homicide might shatter assumptions people have about themselves, others and the world around them, giving rise to feelings of injustice, unfairness, guilt, anger, anxiety, and revenge (14, 19). This may be especially true for those who lost someone to whom the attachment was especially strong, such as a child or spouse (5). Thirdly, in the immediate aftermath of a homicide, additional stressors associated with police investigations, trial procedures and media coverage may fuel distress (20). Although feelings of anger and revenge might not diminish after the conviction of the perpetrator (21), an ongoing trial, unsentenced trials, and dissatisfaction about the criminal justice are associated with higher PTSD scores and a complicated grieving process (19, 21, 22). Fourthly, survivors’ relationships with friends, family, and the community can become strained, as they might face stigmatization, social withdrawal, and isolation, thereby alienating them from society (10, 11, 22). In the United Kingdom (UK), Victims’ Commissioner Casey was appointed by the Ministry of Justice (MoJ) to promote the interests of victims and witnesses of crimes. In her review on the needs of families bereaved by homicide, she found that that separation, forced relocation, job loss, and financial difficulties commonly occurred (10).

Although the emotional consequences of homicidal loss evidently merit clinical attention, options for preventive and curative treatments are scarce. In existing literature on the treatment of traumatic loss, grief- and trauma-focused interventions are described, as well as interventions integrating working mechanisms of both (23, 24). For the treatment of PTSD, trauma-focused cognitive behavioral therapy (CBT) and Eye Movement Desensitization and Reprocessing (EMDR) are implemented in treatment guidelines (25). Furthermore, social work and family-focused therapy are recommended (19). Due to the aforementioned complexities surrounding a homicide, homicide survivors constitute a special group warranting a standardized treatment tailored to their specific needs. However, few well-researched options for treatment exist. One study found EMDR and CBT to be promising treatments for homicidally bereaved individuals (25), but their sample was characterized by a large treatment delay. It is therefore of importance to fortify the evidence base of existing treatments and to investigate their effectiveness over different samples.

In the UK, the attention toward the needs of homicide survivors increased strongly after publication of Casey’s report (12). It was concluded that homicide survivors constitute a vulnerable group that did not receive the support they needed. In 2010, ASSIST Trauma Care, a specialist Not-for-Profit Organization offering specialized therapeutic services to those affected by psychological trauma, was contracted through the government to deliver this support.

### ASSIST Homicide Bereavement Therapeutic Service—A Model of Care

Upon request by the homicide survivor or recommendation by Victim Support or other homicide support agencies that provide practical, judicial, and emotional support to homicide survivors, homicide survivors can be referred to ASSIST Trauma Care. Upon first contact, ASSIST Trauma Care provides information about their services and performs a first diagnostic assessment. Then, based on this assessment and the client’s needs, clients are offered (a) so termed “early intervention”, consisting of one to five face-to-face sessions oriented on psycho-education and coping strategies only, or b) face-to-face traumatic-grief focused CBT (TG-CBT). The current study focuses on the TG-CBT only. The TG-CBT consists of approximately 15 sessions in which psycho-education, exposure on the traumatic loss, writing exercises, giving meaning, and activation are covered. Although the TG-CBT is tailored to the client’s needs, it follows a systematic approach and includes the same therapeutic ingredients for all patients. These include an initial 15 sessions that can be extended depending on individual circumstances, the duration of legal and court proceedings and higher complexity of the case; implementation of evidence-based trauma-focused CBT [National Collaborating Centre for Mental Health (NICE) (26)] and grief-focused CBT (26–28); provision of an outreach model of care with the sessions taking place at the office of ASSIST or at the homes of the clients; individual sessions with possibilities of involvement of supporting others; deployment of qualified and experienced therapists receiving regular training and supervision (from the Oxford Cognitive Therapy Centre); and close collaboration with caseworkers of Victim Support to provide practical and judicial support throughout the process.

### Purpose of the Present Study

The first aim of the current retrospective study was to offer a preliminary evaluation of the potential effects of a traumatic grief-focused CBT in terms of reductions in symptoms of posttraumatic stress (PTS), prolonged grief (PG), depression and anxiety, and general functional impairment, based on data of over 900 people. We also investigated whether age, gender, being a direct witness to the homicide, closeness to the victim, recentness of the loss, status of the funeral and verdict, duration of therapy, and number of missed sessions were associated with changes in symptoms following treatment. The second aim was to examine whether baseline symptom severity was related to the aforementioned characteristics.

Concerning our first aim, we hypothesized that PTS, PG, depression, and anxiety symptom severity and general functional impairment would be significantly lower at the end of treatment compared to the start of treatment. We tentatively expected that higher symptom severity at the start of treatment would be associated with larger symptom reduction during treatment. We did not expect to find any effects for gender, age and recentness of the loss on symptom change during treatment (26). Because of the exposure-oriented nature of the therapy, we expected clients who were direct witnesses to the homicide to show a larger reduction in PTS, PG, and anxiety symptoms than those who were not. Being a parent or partner of the victim was expected to be unrelated to the magnitude of symptom reductions (29). We expected the funeral being held or verdict being spoken during therapy or not at all rather than before therapy would result in smaller reduction of PTS and PG symptoms during treatment; and that clients with a so termed “history of mental health problems”, defined as previous mental health treatment and/or a history of abuse (30), would benefit less from therapy as demonstrated by a smaller reduction of all symptoms. Finally, we expected no association for therapy duration or number of missed sessions with change in symptoms during treatment.

For our second aim, we hypothesized that female gender, younger age, being a direct witness to the homicide, having a closer relation to the victim (being a parent or partner to the victim) and the funeral or trial not yet have taken place would negatively influence symptom severity at the start of treatment (21, 31). Since clinical PTSD and PGD levels following violent loss have been shown to remain relatively stable over time without treatment, we expected that baseline symptom severity would not be associated with the time that had passed since the loss occurred (13, 30). We expected that clients with higher baseline symptoms would require more TF-CBT sessions but also miss more TF-CBT sessions.

As suggested by previous research, many factors may influence the expression of symptoms and the effects of treatment following bereavement by homicide (7, 11, 32). To the knowledge of the authors, the current study is unique in that it evaluates a systematic treatment approach specifically tailored to the needs of homicide survivors, in terms of changes in various outcomes (including symptoms of PG, PTS, depression, and anxiety and general functioning) in a sample that is large enough to explore potential moderators of treatment effects (i.e., differences in treatment effects for different subgroups). The present study adds to existing research in a number of ways. First, this study contributes to the overall understanding of the manifestation and prevalence of a variety of mental health problems following a homicide, building on previous inconclusive research in which various and smaller samples were used. Secondly, by describing the effects of a traumatic-grief focused outreaching model of care, it can inform the development of guidelines for psychosocial care tailored to the needs of homicide survivors. Finally, the current study explores whether the possible effects of the intervention differ as a function of several sociodemographic, homicide-related, and therapy-related characteristics, which may further add to the development of such guidelines.

## Method

### Study Design

The present study was designed as a retrospective, naturalistic pretest-posttest study among homicide survivors who received and completed traumatic grief focused cognitive behavioral treatment through the services of ASSIST Trauma Care.

### Participants

Between July 2010 and March 2017, 2,042 persons were referred to ASSIST following bereavement by homicide. After referral and initial contact, 607 persons (29.7%) decided not to engage. Reasons for this varied from time issues to not wanting to live through the traumatic experience, to already being engaged in a therapy elsewhere (33). The so termed “Early intervention” was offered to 330 persons (20.9%). The remaining 1,105 persons (54.1%) engaged in the full course of TG-focused therapy (CB-CBT) evaluated in the current study. For the purpose of the study, 146 children below the age of 18 years (13.2%) were excluded from analyses, leaving 941 adults. Of these, 12 (1.3%) did not complete any of the questionnaires at baseline nor at the end of therapy and were therefore also excluded from analyses. The final sample used in the current study consists of 929 adults, all of whom completed therapy by March 2017 and completed at least one baseline measure.

### Ethical Standards

The current collaborative study with ASSIST Trauma Care was approved by the Ethics Committee of the Faculty of Social and Behavioral Sciences of Utrecht University and conducted in accordance with ethical guidelines from the UK for service evaluation. Written consent was obtained from all participants at the start of treatment. All information from the clients’ files was anonymized and entered into a dataset that was made accessible only to the researchers and stored on a secured server.

### Procedure

Questionnaires to measure symptoms of PTSD, PGD, depression, and anxiety and functional impairment were administered at intake and at the end of treatment. Information about demographics, the homicide, judicial procedures and whether or not the client had a history of mental health problems was documented in the client’s files. In 2010, only symptoms of PTSD were measured. Later, the additional symptoms were added to the test battery to provide a more complete picture of the effects of the service.

### Measures

#### Predictors

Sociodemographic variables included age and gender. Homicide-related information included relationship to the deceased, which was originally categorized into 22 categories. To simplify the analyses, we dichotomized this variable into having lost a partner or child rather than someone other than a partner of child (0 = No, 1 = Yes). Furthermore, dichotomized variables indicated whether or not the client witnessed the homicide (0 = No, 1 = Yes); if, as compared to the funeral taking place before therapy, the funeral took place during therapy (0 = No, the funeral took place before therapy, 1 = Yes, the funeral took place during therapy) or had not (yet) taken place (0 = No, the funeral took place before therapy, 1 = Yes, the funeral has not yet taken place); if, as compared to a verdict being spoken before therapy, a verdict was spoken during therapy (0 = No, a verdict was spoken before therapy, 1 = Yes, a verdict was spoken during therapy), or had not (yet) been spoken (0 = No, the verdict was spoken before therapy, 1 = Yes, the verdict has not yet been spoken); and whether or not the loss had occurred less than six months ago (0 = No, 1 = Yes). For each client, clinicians indicated whether or not they had a so-termed “history of mental health problems” referring to previous mental health treatment and/or a history of abuse (0 = No, 1 = Yes). Lastly, based on the client files, we gathered information on therapy-related variables including duration of therapy in hours and number of missed sessions.

#### Impact of Event Scale (IES)

Symptoms of posttraumatic stress were measured by the IES (34). This self-report questionnaire includes 15 items rated on 4-point scales ranging from 0 (not at all), 1 (rarely experienced), 3 (sometimes experienced), to 5 (often experienced), the summation of which provides an index of traumatic stress. A score of 26 was used as a cut-off for clinically relevant scores (23, 34). The scale has sound psychometric properties (34). In the present study, reliability was acceptable for the total scale (α = .78).

#### Inventory of Complicated Grief (ICG)

PG symptoms were measured by the 19-items self-report ICG. (35). Items are rated on 5-point scales ranging from 0 (never) to 4 (always). A cut-off score of 26 was used to indicate a probable PGD (35). The ICG has shown high internal consistency, test-retest reliability, and concurrent validity (35). In the current study, Cronbach’s alpha was good (α = .86).

#### Patient Health Questionnaire (PHQ-9)

Self-reported symptoms of depression were measured using the 9-item PHQ-9 (36). Items are rated on 4-point scales ranging from 0 (not at all) to 3 (nearly every day) with higher scores indicating more severe depression. A score of 10 or higher indicates a moderate level of depression (37). The PHQ-9 is a reliable and valid measure (36). In the current study, Cronbach’s alpha was good (α = .85).

#### Generalized Anxiety Disorder Scale (GAD-7)

The GAD-7 (38) is a short self-report questionnaire that measures symptoms of anxiety. The questionnaire includes seven items. Each item can be scored on 4-point scales ranging from 0 (not at all) to 3 (nearly every day). A score of 10 or higher indicates a moderate level of anxiety (38). Evidence supports the reliability and validity of the GAD-7 (39). In the current study, Cronbach’s alpha was good (α = .84).

#### Work and Social Adjustment Scale (WSAS)

With the WSAS (40), the degree of impaired functioning attributable to an identified disorder can be measured. The self-report scale contains five items referring to several facets of life. Items are scored on scales ranging from 0 (not at all) to 8 (very severely), indicating how much a person’s problems affect their ability to carry out certain activities. The WSAS has good psychometric properties (40). Cronbach’s alpha in this sample was good (α = .81).

### Data Analyses

Descriptive analyses were performed and assumptions were checked using SPSS [version 25.0; (63)]. Frequencies, means and bivariate and polychoric correlations were calculated for all variables, the latter one using Mplus version 7 (41). Dropout analyses were performed by comparing clients with posttreatment scores with clients with no posttreatment scores using one-way analyses of variance for continuous variables and chi-square tests for categorical variables. We used Latent Growth Modeling, a statistical technique used in structural equation modeling (SEM), to analyze our repeated measures data and to classify individuals into distinct groups based on individual response patterns. LGM was conducted with Amos [version 19.0; (42)]. Missing data were handled using full information maximum likelihood (FIML) estimation (43). With FIML, a likelihood function for each individual is estimated based on the variables that are present so that all the available data are used. Literature indicates that the bias in FIML parameter estimates is relatively unaffected by the amount of missing data (44). In SPSS, missing data were handled using pairwise deletion. At baseline, 6.9% of scores on the IES were missing, and 44.6%, 21.3%, 20.6%, and 53.2% of scores on the ICG, PHQ, GAD, and WSAS, respectively. At the posttreatment assessment, 13.5% of scores on the IES were missing, and 41.1%, 24.2%, 22.7%, and 54.7% of scores on the ICG, PHQ, GAD, and WSAS, respectively. The large amount of missing data is explained by the gradual addition of other questionnaires to the IES to measure a broader range of mental health complaints than just PTS symptoms. Due to this reason, the values are considered to be missing at random. Despite the variation in sample size on the outcome measures, the effect size for these analyses are expected to exceed Cohen’s convention for a small effect (d = .20) with a power of.80 (45).

**Figure 1:**
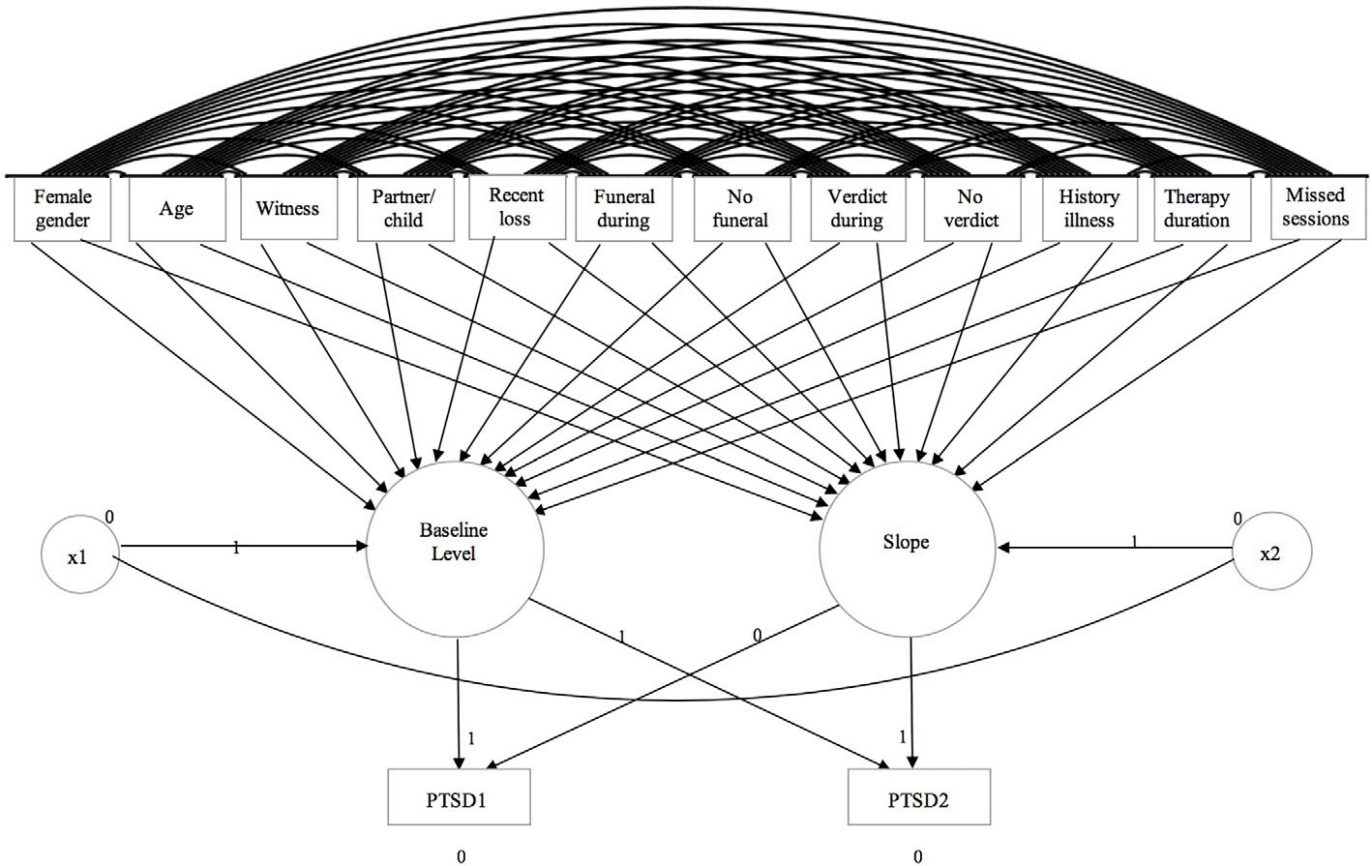
Example of the latent growth model with PTS symptoms as outcome.

An example of a latent growth model for PTS symptoms is displayed in **Figure 1**. In total, five latent growth models were specified, each comprising (1) baseline levels of PG, PTSD, depression, and anxiety and functional impairment as well as (2) change during treatment in these symptoms and functional impairment as the two latent factors. The pre- and posttreatment scores on these measures were specified as the indicators. For the baseline level factor, paths with regression weights fixed to one were drawn to pre- and posttreatment scores, while for the change during treatment factor, only a path was drawn to posttreatment scores with regression weight fixed to one. Residual error variances of the indicators were fixed at zero to enable the model to be estimated. The model was saturated, implying a perfect fit. This was deemed appropriate in light of our aims, as we sought to deploy a model that allowed us to explore the size, direction, and statistical significance of direct and indirect path coefficients of all possible effects. Hence, no model fit indices for the model are given. This model fits prior research using a comparable sample and design (46). For the calculation of Cohen’s *d* (47), we used the mean and standard deviation of the change in symptoms during treatment and the correlation between pre- and posttreatment symptoms for all measures (48). To each of the five models, all independent predictors were specified to both factors. Standardized coefficients were calculated to be able to evaluate the relative strength of each independent variable in predicting the dependent variable. Squared multiple correlations were calculated to estimate the percentage of explained variance by the model.

## Results

### Descriptive Statistics

Descriptive data of sociodemographic, homicide-related, history of mental illness, and therapy-related variables of the complete sample consisting out of 929 clients are presented in **Table 1**.

**Table 1:** Sociodemographic characteristics, homicide characteristics, history of mental health problems and therapy characteristics at baseline.

Variable	*M*	*SD*	*n*	*%*	*n*obs	*%n*
Sociodemographic						
Female gender			700	75.4	929	100.0
Age	43.46	14.45			817	87.9
Homicide related						
Witness			178	19.2	928	99.9
Child/spouse			485	52.2	929	100.0
Recent loss (≤ 6 months)			470	50.6	915	98.5
Funeral before therapy			860	92.9	926	99.7
Funeral during therapy			53	5.7	926	99.8
No funeral			13	1.4	926	99.7
Verdict before therapy			304	32.7	810	87.2
Verdict during therapy			270	29.1	810	87.2
No verdict			236	25.4	810	87.2
History of mental health problems			367	39.5	927	99.8
Therapy related						
Therapy duration (hours, range: 3–59)	16.46	7.60			928	99.9
No. of missed sessions	1.74	2.26			927	99.9

nobs, number of observations; %n, percentage of total number of observations.

Individuals for whom posttreatment scores were unavailable, for whatever reason (e.g., ended therapy preliminary, or finished therapy but were unable to fill out the end measures), were considered study-dropouts. Patients who completed posttreatment measures (N = 859) did not differ from study-dropouts (N = 70) in terms of gender, age, being a direct witness to the homicide, recentness of the loss, verdict status, history of mental health problems, or any of the therapy-related variables. However, study-dropouts were more likely than completers to have lost a child or spouse (40.0% vs. 28.6%, *p* = .040), having had a funeral during therapy instead of before therapy (13.0% vs. 5.1%, *p* = .006) and not having had a funeral yet instead of having had a funeral before therapy (4.3% vs. 1.2%, *p* = .030).

### Aim 1: Treatment Effect Sizes and Predicting Change During Treatment

**Table 2** shows symptom severity as measured with the IES, ICG, PHQ, and GAD and functional impairment as measured with the WSAS; percentages of people scoring above the cutoff at the start and end of treatment; and the change in symptoms during treatment plus Cohen’s d effect sizes. All symptoms and functional impairment reduced significantly and effect sizes were all high.

**Table 2:** Mean symptom severity and functional impairment at start and end of treatment and change in symptoms and functional impairment during treatment.

	Start of treatment					End of treatment					Change during treatment		
	*M*	*SD*	*%*cut	*n*	*%n*	*M*	*SD*	*%*cut	*n*	*%n*	*M*	*SD*	*d*
IES	50.37	13.55	95.1	876	94.3	21.21	16.36	33.2	814	87.6	29.25***	17.62	1.94
ICG	43.45	13.93	89.0	521	56.1	23.64	15.38	40.2	554	56.6	19.57***	14.37	1.33
PHQ	15.87	6.83	58.6	741	79.8	6.50	6.57	12.9	713	76.7	9.39***	6.76	1.40
GAD	13.95	5.54	76.8	747	80.4	6.10	5.38	21.6	727	78.3	7.83***	6.12	1.43
WSAS	20.83	10.23	52.2	440	47.4	9.06	8.69	12.6	426	45.6	12.04***	8.98	1.26

IES, Impact of Events Scale; ICG, Inventory of Complicated Grief; PHQ, Patient Health Questionnaire; GAD, General Anxiety Disorder; WSAS, Work and Social Adjustment Scale; %cut, percentage of scores above cut-off score; %n, percentage of total number of observations. Baseline and change symptoms based on FIML and end symptoms based on available data. ***p <.001.

**Table 3** shows the associations between sociodemographic, homicide-related, and therapy-related characteristics on the one hand and the change of symptoms and functioning during treatment on the other hand. The intercept and slope of all measures correlated significantly and negatively. This indicates that clients with higher baseline symptom severity showed a larger decline in symptoms during treatment than those with lower baseline symptom severity.

**Table 3:** Associations of demographic and homicide characteristics, history of mental health problems, and therapy characteristics with symptoms of PTSD, PGD, general anxiety, depression, and functional impairment at baseline and change during treatment.

Variable	IES		ICG		PHQ		GAD		WSAS	
	Baseline*B*	Slope*B*	Baseline*B*	Slope*B*	Baseline*B*	Slope*B*	Baseline*B*	Slope*B*	Baseline*B*	Slope*B*
Sociodemographics										
Female gender	0.17***	−0.06	0.17***	−0.04	0.18***	−0.05	0.18***	−0.06	0.16***	−0.10*
Age	−0.11**	0.07	−0.11*	0.09	−0.01	0.02	−0.08*	0.07	0.01	−0.05
Homicide related										
Witness	0.05	−0.03	0.01	−0.04	0.06	−0.09*	0.04	−0.09*	0.00	−0.01
Child or spouse	0.07	0.04	0.16***	0.05	0.09*	−0.01	−0.02	0.05	0.15**	−0.01
Recent loss (≤ 6 months)	0.11**	−0.06	0.09	−0.13*	0.05	−0.03	0.04	−0.05	0.04	−0.05
Funeral during therapy	−0.07*	0.04	−0.07	0.04	−0.03	0.01	−0.05	0.01	−0.01	0.02
No funeral	0.01	0.02	−0.13***	0.11*	−0.02	0.04	−0.05	0.03	−0.02	0.08
Verdict during therapy	−0.03	0.10*	0.02	0.13*	−0.04	0.08	0.02	0.04	0.12*	−0.08
No verdict	−0.05	0.11*	0.03	0.07	−0.05	0.06	−0.04	0.08	0.03	0.03
History of mental health problems	0.09**	0.22***	0.17***	0.16***	0.22***	0.15***	0.16***	0.13***	0.09	0.26***
Therapy related										
Therapy duration (hours)	0.10**	0.02	0.12**	0.04	0.11**	0.04	0.09*	0.08*	0.15**	−0.05
No. of missed sessions	0.12***	−0.04	0.08	−0.05	0.11**	−0.08*	0.12***	−0.10*	0.14**	−0.07
Baseline scores	−0.54***		−0.49***		−0.62***		−0.63***		−0.66***	
R^2^	0.12**	0.08	0.18	0.08	0.17	0.04	0.14	0.06	0.16	0.08

B, Standardized regression coefficient (beta weight); IES, Impact of Events Scale; ICG, Inventory of Complicated Grief; PHQ, Patient Health Questionnaire; GAD, General Anxiety Disorder; WSAS, Work and Social Adjustment Scale; R^2^, proportion of variance by the model. *p <.05. **p <.01. ***p <.001.

Age was not related to the change of any of the symptoms; having a history of mental health problems was associated with a smaller reduction of all symptoms; and the verdict being spoken during therapy compared to before therapy was associated with smaller reductions in PTS and PG symptoms.

Female gender was associated with larger improvement in general functioning but not with changes in symptoms. Being a direct witness of the homicide did not have an effect on change in symptoms of PTS or PG, but was, in fact, associated with greater reductions in depression and anxiety symptoms. No funeral being held yet was related to smaller reductions on PG symptoms but was not associated with changes in PTS symptoms. No verdict being spoken was associated with smaller reductions on PTS symptoms but was unrelated with changes in PG symptoms. Recentness of the loss was associated with larger symptom reductions.

The funeral being held during therapy was unrelated to change in symptoms and functioning. In addition, recent loss was associated with larger PG symptom reduction and was not associated with changes of any other symptoms during treatment.

We found a larger decrease in anxiety symptoms for clients who had a shorter therapy duration. Clients who benefitted more from therapy in terms of symptoms of anxiety and depression tended to miss more sessions.

### Aim 2: Predicting Symptoms at Baseline

Data on the extent to which symptoms of PTS, PG, depression, and anxiety and functional impairment varied as a function of the sociodemographic, homicide-related, and therapy-related variables are summarized in **Table 3**.

Female gender was associated with higher baseline symptom severity on all measures. Younger age was associated with higher baseline symptom severity except for depression symptoms and functional impairment; and having a history of mental health problems was associated with higher baseline symptom severity but not with higher functional impairment. Having lost a child or spouse was associated with higher baseline PG and depression symptom severity and with higher functional impairment, but not with other symptoms. Recent loss was associated only with higher baseline PTS symptom severity.

Being a direct witness to the homicide was not related to higher baseline symptom severity; the funeral being held before the start of therapy compared to during therapy was associated with higher baseline PTS symptom severity while the funeral being held before therapy compared to the funeral not being held at all was associated with higher PG symptom severity; and the timing of the verdict showed no association with baseline symptom severity.

Clients with higher baseline symptom severity tended to have a longer therapy duration and miss more sessions.

**Table 3** shows the relative strength of the association between the sociodemographic, homicide-related, history of mental illness, and therapy-related predictors; and baseline symptom severity and symptom reduction during treatment, as indicated by their standardized coefficients and their significance; and the proportion of variance explained by all the predictors on baseline symptom severity and symptom reduction during treatment.

## Discussion

The current study introduced and evaluated the effects of an outreaching model of care following bereavement through homicide and examined whether baseline symptom severity and functional impairment and their changes during treatment varied as a function of sociodemographic- and homicide-related characteristics, having a history of mental health problems and treatment-related characteristics. In total, 929 treatment receiving homicide survivors were included. Prevalence of severe symptoms across disorders and general functional impairment at baseline was high, adding to the body of literature describing the broad range of detrimental effects of homicide on the mental health of homicide survivors [e.g., (13)]. Altogether, the decrease in symptoms across disorders and functional impairment following treatment suggests the potential effectiveness of the treatment. For change in symptom severity and functional impairment during treatment, having a history of mental illness was the only consistent predictor across all outcomes. Being a direct witness was predictive of change in depression and anxiety symptoms and the status of the verdict was predictive of change in PG and PTS symptoms.

Having a history of mental illness, being younger of age and female and having lost either a child or spouse consistently predicted higher symptom severity and functional impairment at baseline.

The first aim of the study was to introduce a traumatic grief-focused outreaching model of care and to provide a preliminary evaluation of its potential effectiveness in terms of reduction on symptoms of PTS, PG, depression, and anxiety and functional impairment. As shown by the LGM, scores on all measures decreased significantly and with a large effect size, thereby confirming our first hypothesis. This cautiously implies that the current model of care as employed by ASSIST Trauma Care might be effective in reducing a broad range of mental health complaints in treatment receiving homicide survivors, while overcoming the geographic, financial or waiting-list barriers for care as reported by the Victims Commissioner (10). Consequently, that model of care could serve as a base to develop international guidelines for the treatment of homicide survivors. The current model includes several specific components that merit attention, namely, close collaboration between primary care (e.g., Victim Support) and specialized care (e.g., organizations offering TGF-CBT), access to timely assessment, access to TGF-CBT that is free of charge, availability of care across the whole country, and ongoing training and supervision of caretakers offering the TGF-CBT, specific to the complexities of the context and aftermath following a homicide.

Furthermore, we examined whether change in symptom severity and baseline symptoms varied as a function of sociodemographic variables, characteristics of the homicide, and having a history of mental illness. Importantly, the variable “history of mental illness” was most strongly and consistently associated with change in symptoms and baseline symptoms. Given the large sample size of the current study, this provides strong indications that previous experiences such as a history of mental health problems or abuse might be an important determinant for poorer response to psychotherapeutic care and the development of a variety of complaints after bereavement following a homicide.

Corresponding to the findings of Boelen, van Denderen, and de Keijser (5) who found that feelings of anger and revenge did not diminish according to the progress of the judicial process, baseline symptom severity did not differ as a function of verdict status in the current sample. However, additional or secondary stressors following traumatic loss may negatively influence the course of complaints and obstruct recovery. Our findings of smaller reductions of PGD and PTS symptoms implicate that a slow course of justice or the absence of a verdict might negatively impact treatment response. This underlines the impact of the judicial process on the therapeutic outcome and the importance of close collaboration between case workers and the therapists to provide additional information and support regarding the course of justice.

Partly in par with our expectations, except for PTS symptoms, baseline symptom severity and functional impairment were unrelated to recentness of the loss. This strengthens previous findings that distress following homicide does not naturally decrease over time (13, 30). We also found that homicide survivors whose loss occurred recently displayed a greater decline in PG symptoms, suggesting that offering intervention in the early months following loss may actually be beneficial for a subgroup experiencing severe distress (49, 50). In fact, these findings support the public health model of bereavement support that differentiates between low, moderate, and high risk and need for support following bereavement (51). Considering bereavement by homicide as a high-risk category, immediate referral to specialist care is desirable.

Homicide survivors witnessing the homicide did not display higher symptom severity at baseline compared to those who were not a direct witness. This accords with the findings of Hertz, Prothrow-Stith, and Chery (9), based on a literature search on homicide victims, showing that witnesses and non-witnesses display similar symptom severity; and the notion that intrusive images similar to those of direct witnesses may be evoked by reconstructive fantasy based on police and witness reports (18, 52, 53). Lastly, closer kinship to the deceased did not have a significant relation with baseline PTS symptom severity, suggesting that PTS symptoms are likely to develop following a homicide regardless of closeness to the victim. This may be explained by the violent and horrific nature of the event itself. Corroborating the finding of a high risk bereavement profile following such a loss (51), having lost a partner or spouse was actually strongly associated with higher baseline PG symptom severity in the current study. However, we did not measure quality of the relationship with the deceased [cf. (54)] and it would be of interest to do so in future research.

Finally, as expected, clients with more severe baseline symptoms eventually underwent more sessions, which is in accordance with prior findings that patients with more severe and complex symptoms receive treatments that last longer (55). Somewhat unexpectedly, however, clients who benefitted more from therapy in terms of declines in anxiety and depression also tended to miss more sessions. One possible explanation for this finding might be that when clients feel they benefit from therapy, they are less strongly motivated for the treatment and more inclined to skip the last sessions. However, an alternative explanation may be that patients who had fewer sessions engaged in less exposure, allowing them to avoid confrontation with the acceptation of the loss [cf. (56)].

The findings of the current study may have several practical implications. One key message for “stakeholders” of homicidal bereavement (e.g., mental health care workers, policy makers, and police and court officials) is that homicide survivors are likely to develop a broad range of severe mental health complains that can be successfully alleviated by offering timely TG-CBT treatment. Early assessment, extra sessions and application of additional interventions parallel to CBT (e.g., group therapy) may prove beneficial for individuals with a history of mental health problems. The occurrence of the funeral and verdict may temporarily increase symptoms, pointing toward the possible necessity of additional sessions to ensure therapeutic holding during those events, as well as the importance of prompt handling of the judicial process. Since the status of the funeral was unrelated to therapy outcome, it might not be necessary to postpone an intervention to after the funeral. Lastly, therapists might consider that exposure therapy may be as beneficial for those who did not witness the crime as for those who did. Concluding, it may be of importance to coordinate treatment with other parties involved so that it can be tailored to the intertwinement between the complex external circumstances and the needs of the individual at that time.

### Limitations and Strengths

Some limitations of the current study should be taken into consideration. First of all, because this study did not include a control group, we were unable to evaluate the effects of TGF-CBT relative to other treatment or no treatment and natural recovery. Secondly, the use of self-questionnaires prohibits any statements about the presence of disorders to be made and may lead to an overestimation of symptom severity (57). Prevalence rates in the current study were indeed higher than those in other studies (14, 21, 58, 59). Because this study focused on help receiving individuals, this might be attributable to a selection bias as people with low level of complaints were not included, thereby limiting the generalizability. In the same line of reasoning, since help seeking has been demonstrated to discriminate between those who benefit most and those who do not (60), the decline in scores may be positively biased. Conversely, the later addition of questionnaires other than the IES to the test battery may underestimate the prevalence of symptoms other than PTS symptoms. Additionally, as only two time points were used in the LGM, no statements could be made about the shape of growth of symptoms over time. Lastly, in aiming to describe the current service-delivery approach to inform international guidelines, we did not unravel which specific elements of the therapy were effective, such as cognitive restructuring, exposure, the outreaching nature of the therapy or the practical support as provided by Victim Support. Notwithstanding these considerations, the results of this study strengthen and quantify previous findings on grief interventions using smaller sample sizes and various specific subgroups that are difficult to generalize. This study is the first to address a multitude of mental health complaints after bereavement through homicide in such a large clinical sample while considering predictors related to the homicide, having a history of mental illness, sociodemographic information, and the therapy.

### Implications for Future Research

Future studies should include a control group and follow-up measurements in order to determine the effectiveness and endurance of the current model of care on PTSD, PGD, depression, and anxiety disorders. Prospective, longitudinal studies are needed to examine the causal direction between the predictors used in this study and the development of symptoms of PTS, PG, depression, and anxiety following homicide, preferably including more than two waves so individual patterns in changes of symptoms can be explored.

Concerning generalizability, it would be of interest to explore whether the current service-delivery approach as offered by ASSIST could be implemented in other countries and cultures with similar effects and to examine whether the current model could be beneficial for other populations facing bereavement and/or traumatic experiences. Lastly, it would be of great interest to explore whether clearer patterns of predictors will emerge when examining the co-occurrence of these symptoms or symptom profiles, using latent class and latent trajectory analyses. In conclusion, the service-delivery approach described in the current study appears to be effective in reducing mental health complaints following bereavement through homicide. Having a history of mental illness was a strong predictor for reaction to treatment. The results furthermore provide insight in the severe and lasting detrimental mental health outcomes that may arise following homicidal loss and provides evidence that timely, outreaching, specialized treatment can be beneficial to counteract these effects.

## Data Availability Statement

The datasets generated for this study will not be made publicly available because the data used in the current study contains information from a patient population.

## Ethics Statement

The current collaborative study with ASSIST Trauma Care was approved by the Ethics Committee of the Faculty of Social and Behavioral Sciences of Utrecht University and conducted in accordance with ethical guidelines from the UK for service evaluation. Written consent was obtained from all participants at the start of treatment.

## Author Contributions

BG developed the intervention, and together with RW collected, managed, and entered the data. GS and SS designed the study, analyzed the data, and together with PB did the writing. All authors contributed to the article and approved the submitted version.

## Conflict of Interest

The authors declare that the research was conducted in the absence of any commercial or financial relationships that could be construed as a potential conflict of interest.
