# Corrigendum: The UK National Homicide Therapeutic Service: A Retrospective Naturalistic Study Among 929 Bereaved Individuals

**DOI:** 10.3389/fpsyt.2021.668178

**Published:** 2021-06-10

**Authors:** Suzan Soydas, Geert E. Smid, Barbara Goodfellow, Rachel Wilson, Paul A. Boelen

**Affiliations:** ^1^Department of Clinical Psychology, Utrecht University, Utrecht, Netherlands; ^2^ARQ Centrum'45, ARQ National Psychotrauma Centre, Diemen, Netherlands; ^3^University of Humanistic Studies, Utrecht, Netherlands; ^4^ASSIST Trauma Care, Rugby, United Kingdom

**Keywords:** homicide, bereavement, loss, grief, posttraumatic stress disorder, bereavement interventions, psychological interventions

1. In the original article “Djelantik AAAMJ, Smid GE, Kleber RJ, Boelen PA. Symptoms of prolonged grief, post-traumatic stress, and depression after loss in a Dutch community sample: a latent class analysis. *Psychiatry Res*. (2017) 247:276–281.” and “Lenferink LIM, van Denderen M, de Keijser J, Wessel I, Boelen PA. Prolonged grief and post-traumatic stress among relatives of missing persons and homicidally bereaved individuals: a comparative study. *J Affect Disord*. (2017) 209:1–2. doi: 10.1016/j.jad.2016.11.012” were not cited in the article. The citations have now been inserted in **“*Introduction,” “Paragraph 1”*** and should read:

“In response to the death of a loved one, many people experience acute emotional distress and symptoms of mental health disorders, which generally decrease over time. However, for a significant minority, these symptoms may persist and develop into psychopathology, such as post-traumatic stress disorder (PTSD), major depressive disorder (MDD), anxiety disorders and prolonged grief disorder (PGD) [e.g., (1, 2)].”

2. In the original article “IBM Corp. *IBM SPSS Statistics for Windows, Version 25.0*. Armonk, NY: IBM Corp (2017).” was not cited in the article. The citation has now been inserted in **“*Method,”* “Data analyses,” “*Paragraph 1”*** and should read:

“Descriptive analyses were performed and assumptions were checked using SPSS [version 25.0; (43)].”

The authors apologize for this error and state that this does not change the scientific conclusions of the article in any way. The original article has been updated.

## References

[1] Djelantik AAAMJ Smid GE Kleber RJ Boelen PA. Symptoms of prolonged grief, post-traumatic stress, and depression after loss in a Dutch community sample: a latent class analysis. Psychiatry Res. (2017) 247:276–81. 10.1016/j.psychres.2016.11.023 27936439

[2] Lenferink LIM van Denderen M de Keijser J Wessel I Boelen PA. Prolonged grief and post-traumatic stress among relatives of missing persons and homicidally bereaved individuals: a comparative study. J Affect Disord. (2017) 209:1–2. 10.1016/j.jad.2016.11.012 27865122

[43] IBM Corp. IBM SPSS Statistics for Windows, Version 25.0. Armonk, NY: IBM Corp (2017).

